# A Functional Architecture of Optic Flow in the Inferior Parietal Lobule of the Behaving Monkey

**DOI:** 10.1371/journal.pone.0000200

**Published:** 2007-02-07

**Authors:** Milena Raffi, Ralph M. Siegel

**Affiliations:** Center for Molecular and Behavioral Neuroscience, Rutgers University, Newark, New Jersey, United States of America; University of Southern California, United States of America

## Abstract

The representation of navigational optic flow across the inferior parietal lobule was assessed using optical imaging of intrinsic signals in behaving monkeys. The exposed cortex, corresponding to the dorsal-most portion of areas 7a and dorsal prelunate (DP), was imaged in two hemispheres of two rhesus monkeys. The monkeys actively attended to changes in motion stimuli while fixating. Radial expansion and contraction, and rotation clockwise and counter-clockwise optic flow stimuli were presented concentric to the fixation point at two angles of gaze to assess the interrelationship between the eye position and optic flow signal. The cortical response depended upon the type of flow and was modulated by eye position. The optic flow selectivity was embedded in a patchy architecture within the gain field architecture. All four optic flow stimuli tested were represented in areas 7a and DP. The location of the patches varied across days. However the spatial periodicity of the patches remained constant across days at ∼950 and 1100 µm for the two animals examined. These optical recordings agree with previous electrophysiological studies of area 7a, and provide new evidence for flow selectivity in DP and a fine scale description of its cortical topography. That the functional architectures for optic flow can change over time was unexpected. These and earlier results also from inferior parietal lobule support the inclusion of both static and dynamic functional architectures that define association cortical areas and ultimately support complex cognitive function.

## Introduction

The analysis of self-motion perception utilizes an extensive repertoire derived from many cortical areas [1]. Although the representation of optic flow is common in cortex, the tuning of areas differs with the use of optic flow evolving across cortical areas from perception to action. The integration between ascending and descending input, feedforward and feedback signals within a particular cortical region may vary to match the role for that region of cortex.

The posterior parietal cortex is a crucial link for the integration of visuo-motor signals (e.g. optic flow, attention and oculomotor guidance) [2]–[5]. Lesions in the inferior parietal lobule may lead to deficits in the utilization of optic flow in navigational and spatial tasks even with unimpaired simple motion processing [6], [7].

Areas 7a and dorsal prelunate (DP) lie on the dorsal surface of the inferior parietal lobule; they are contiguous in the most dorsal aspect and not separated by the superior temporal sulcus (STS). The neurological motion defects can in part be described in terms of loss of neurons with appropriate properties [6], [7]. Neurons in area 7a are modulated by the retinotopic position of the optic flow stimulus and by eye position [4]. Two properties appear in 7a optic flow neurons [8]. A class of motion selectivity named flow general neurons (FLO-G) and a class named flow particular neurons (FLO-P). The FLO-G neurons are able to distinguish between particular types of optic flow (i.e. radial vs. rotation), while the flow particular neurons (FLO-P) that are tuned for a particular direction of optic flow (i.e. expansion vs. contraction). Area 7a neurons are also modulated by the behavioral importance of the stimulus [9]–[13], which also appears to occur when viewing optic flow [8].

Topographies are key to understanding how a particular function is organized across an area, as well as providing constraints as to how they can be created from local and intercortical projections. The inferior parietal lobule contains a topography of gain fields, with area 7a representing the lower eye position gain fields and area DP representing upper eye position [14]. A retinotopic topography in the ventral and dorsal portions of area DP and in area 7a provide a foundation for position localization [15], as does a patchy representation of attention [5]. The organization of optic flow processing in inferior parietal lobule is less well understood. Electrical recordings have failed to provide compelling evidence of topography [8]. This lack of a cortical topography for optic flow in the parietal lobe has limited its study to a cataloging of motion properties, with little constraints as to their origin or cellular processes for modulation.

Using optical imaging of intrinsic signals optic flow stimuli were found represented in patches in areas 7a and DP. These optic flow patches were superimposed upon the gain field map. Areas 7a and DP optic flow tuning were correlated; however they both varied over a long period of time.

## Methods

The monkeys used in these experiments were the same as in previous studies. All surgical procedures are identical to those described earlier [5], [14], [15]. In short, a metal chamber with an inner diameter of 20 mm was placed over the right hemisphere in the first monkey (M1R) and a chamber with an inner diameter of 25 mm over the left hemisphere in the second monkey (M2L) at AP-7, ML12 in M1R and AP-13, ML15 in M2L. An artificial dura permitted chronic imaging of the tissue [14]–[17]. The reader is referred to our earlier publications [5], [14], [15] for full MRI reconstructions of the chamber locations. It needs to be stressed that the blood vessel running across the chamber is on the flat surface of the brain and that there is no sulcus under the vessel.

Surgeries were performed in accordance with the guidelines of the National Institute of Health and approved by Rutgers University Institutional Review Board for the Use and Care of Animals. These monkeys were used in earlier studies [5], [14], [15].

### Optical recordings

**Figure 1:**
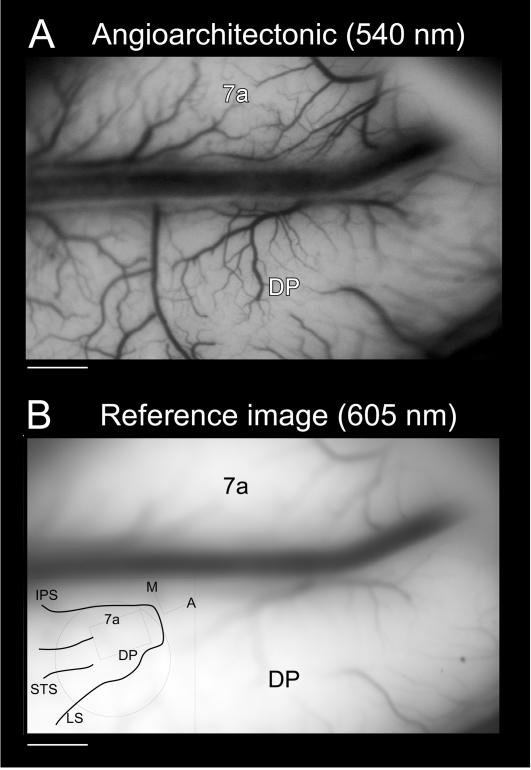
Different wavelength images of cortex. A. Image of the exposed cortex taken with a 540 nm filter (green light) which shows the angioarchitectonic of areas 7a and DP of the left hemisphere of the second monkey (M2L). B. Image of the exposed cortex taken with a 605 nm filter (orange light) during the recording session; the focus is 500 µm below the surface capillaries. The inset of panel B indicates the location of the imaged region for M2L. Note there is an extra unnamed sulcus medial to the STS. Horizontal bars: 1 mm. Data set: M2L 09-24-2002. STS: superior temporal sulcus, IPS: intraparietal sulcus, LS: lunate sulcus, M: medial, A: anterior.

Optical measurements were performed using a modified macroscope consisting of a Nikkor 60 mm macrolens and a 50 mm Nikon 1.2 lens providing a long working distance [5], [14], [15]. Optical recordings were performed daily and before starting recording, an image of the exposed cortex was taken with a 540 nm filter in order to record the angioarchitectonics of areas 7a and DP (Fig. 1A). Images were collected with a 605 nm filter on a halogen illuminator and a stable DC light source (Fig. 1B) at a depth of 500 µm below the surface capillaries [14], [16], [17]. Images were collected at 740×480 pixels and binned online to 370×240 pixels with 18–23 µm/pixel resolution. These data were not temporally or spatially filtered further.

### Behavioral task

**Figure 2:**
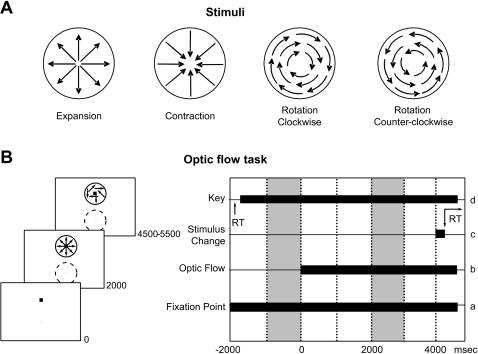
Behavioral protocol. A. Stimuli. Optic flow patterns were made by 128 moving dots with an average speed of 6°/sec. B. Temporal sequence of the behavioral task. At the onset of the fixation point (a) the monkey has to pull a lever (d). After two seconds the optic flow stimulus appears (b) and remains displayed for at least four seconds, after which, at a random time, the optic flow stimulus changes its motion from structured to unstructured (c). The monkey had to release the lever within a maximum reaction time of 650 msec (d). The two filled epochs indicate the temporal windows used in the analysis. The baseline image is acquired during the simple fixation, i.e. all frames collected between −1000 and 0 msec are averaged together; the stimulus image is acquired between 2000 and 3000 msec. RT: reaction time. The thick circle represents an exemplar stimulus position, while the dashed circle represents a potential stimulus position.

The animals were trained to fixate a 0.1° red square in a reaction time task (Fig. 2). At the onset of the fixation point (Fig. 2B,a) the animals pulled a lever (Fig. 2B,d), then after 2000 msec an optic flow stimulus was presented concentric to the fixation point (Fig. 2B,b). Optic flow stimuli consisted 128 moving dots computed and displayed as in [18]. The approximate linear speed of the dots was 6°/sec, which simulated radial expansion and contraction, and clockwise and counter-clockwise rotation (Fig. 2A). The diameter of the display was 10°; dot size was 0.1°, point life was 52 msec. The dot luminance was 32 cd/m^2^ against a background of 1 cd/m^2^. This earlier study demonstrated that monkeys, similar to human subjects, utilized the global optic flow and did not perform the task using local motion cues. Electrophysiological studies in inferior parietal lobule suggest that global flow is processed there rather than local flow [4], [8], [19]–[22].

By following Anderson and Siegel [18], all flows were constructed to have exactly the same speed profiles. The animals detected a change in the optic flow structure in the time window of 4000 to 5500 msec after the trial began (Fig. 2B,c) and released the lever within a maximum reaction time of 800 msec (Fig. 2B,d). The dot life was 533 msec and the dots flickered asynchronously. Initially the fraction of structure was 1; after the change to the unstructured motion the fraction of structure was 0 [23]. Correct performance was rewarded with 0.1 ml juice.

It is known from prior studies that area 7a is activated by fixation in the lower part of the visual field, while area DP is activated by fixation in the upper part and that there is a weak gain field dependence on the horizontal axis [5], [14]. Thus, this test was performed at two angles of gaze only, (10° above or below the primary position) as a control for the strength of the cortical signal. The eye position was monitored monocularly by an infra-red video system (ISCAN Co, Cambridge, MA) to be within 1° of the fixation point. Larger deviations or behavioral errors terminated the trial. Animal typically performed at 90–98% correct.

### Data collection and analysis

Behavioral data was analyzed by an analysis of variance (ANOVA) of the reaction time. The reaction time was the time taken for the key to be released relative to the instant the change began from structured to unstructured motion. A two-way ANOVA was computed with the optic flow stimulus and the eye position as the independent terms as well as an interaction term (PROC GLM, SAS Co., Durham NC). The probability of the main or interaction effects was computed on single experiments. Typically, the monkey performed from 400 to 600 correct trials; hence there were 50 to 75 correct trials per condition. Factors were taken as significant when there was an effect at *p*<0.05. To assess whether there was a correlation between a specific optic flow stimulus and the behavioral performance, a step-wise regression and a circular regression model has been applied [24].

The reaction time for each trial is given by the equation:
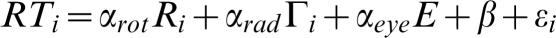
where the “α” terms are slopes of the rotational component *R_i_*, radial component Γ_i_ and eye component *E_i_*, respectively for the *i^th^* trial. The coefficients were computed by a stepwise regression (Procedure STEP in the Statistical Package the statistical package **R** (R Foundation for Statistical Computing, Vienna, Austria http://www.R-project.org.). This regression procedure added or removed coefficients depending on whether the Akaike Information Criterion was improved [5], [14], [15]

The recordings of optical data with the Optical Imaging System Imager 2001 (Rehovot, Israel), were identical to that in earlier papers [5], [14], [15] The collection rate was 7 Hz with a pixel resolution of 370×240; no digital filtering was performed on the images.

After performing a “baseline normalization analysis” with exactly the same timing and trial rejection paradigms as described earlier [5], [14], a simple subtraction analysis across pairs of stimulus conditions was used as to determine the effects of a particular optic flow stimulus and/or a particular eye position. During the baseline period (1000 msec prior to optic flow onset), the monkey was fixating the red square and holding back on the lever. Those effects were subsequently quantified using a regression analysis for representation of optic flow [8], [25], [26]. Each pixel was fit as function of each optic flow stimulus and eye position using the equation:
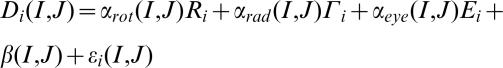
where *D_i_*(*I,J*) is the change in reflectance of the i^th^ trial's for each pixel *(I,J)*, *R_i_* and Γ*_i_* are the rotational and radial conditions respectively with the vertical eye position in degrees (*E_i_*). The three slope coefficients of the regressions [*α_rot_*(*I,J*), *α_rad_*(*I,J*), *α_eye_*(*I,J*)] are for rotation, radial and eye position respectively, β*(I,J)* is the intercept and *ε_i_(I,J)* is the residual error. Because the optical signal is inversed with respect to the neuronal activity, the sign in the model has been corrected by multiplying all parameters by −1 prior to creating the images [14], [17], [27], [28]; hence darker regions would be expected to have the highest rate of neuronal activity.

### Optic flow analysis

The units for the rotation and radial signals require a short comment. A rotational component of “1 unit” refers to a clockwise motion of 6°/sec and a component of “−1 unit” refers to a counter-clockwise rotation of the same speed. Similar definitions are given for radial motion; indeed the radial motion has exactly the same distribution of speeds [18]. Note the units for the motion stimulus are arbitrary; the terms ROT will be used for rotation and RAD for radial. Slope coefficients for the regressions in equations 1 and 2 are thus “msec/ROT” and “%/ROT”, respectively.

The radial and rotational components were represented using a “spiral space” approach [8], [25] in which the rectangular coordinate system of (*α_rot_,α_rad_*) is converted into a polar notation with the angle given as Θ = tan^−1^(*α_rot_/α_rad_*), with adjustment for the appropriate quadrant. For the (I,J) pixel, the angle Θ(*I,J*) represents the steepest slope in the plane defined by “*α_rot_*(*I,J*)*R_i_*+*α_rad_*(*I,J*)Γ*_i_*”. The steepest of the slope is given as the magnitude “||*α_rot_*(*I,J*), *α_rad_*(*I,J*)||”. Angles Θ(*I,J*) were color coded in the functional maps in which each angle represents the specific flow stimulus that maximally activates each pixel (0°, cyan, corresponds to clockwise motion, 90°, blue, corresponds to expansion, 180°, red, corresponds to counter-clockwise and 270°, green, corresponds to contraction). Angles between two orthogonal axes represent an interpolation of the activation due to a combination of two stimuli. Thus a yellow color would represent an interpolated counter-clockwise-contraction spiral.

The spatial frequency and phase components of parameter maps were computed using the analysis in [5]. A region of interest covering the maximal portion of the imaged cortex was selected using a Gaussian mask in order to avoid boundary effects in the Fourier transform (see [5] for additional details). This resulted in 18 one-dimensional cuts through the Fast Fourier Transformation (FFT) space at 10° intervals. The amplitude of the one-dimensional FFT was examined visually to locate the first peak from the 0 cycles/mm center point, representing the lowest spatial frequency in the image. Because the location of the peak slightly varied across sections, the one-dimensional FFT for the vertical orientation (90°) was selected as a model of the data. The peak location was computed in each section and the location of the first peak was used to find its location in the 90° section. The phase as a function of spatial frequency for this orientation was also recorded.

## Results

A total of 38 experiments were performed in two monkeys, 22 in M1R and 16 in M2L. Data were collected for a period of two months in both monkeys. Recordings in M1R had an interruption of three months in the middle of the recordings. Five optical experiments did not show topography of optic flow (three in M1R and two in M2L); those experiments were discarded from the data set. These inaccurate maps are likely due to either an under- or over illumination of the cortex or to an incorrect focus under the cortical surface. All optical data analyses have been performed on the remaining 33 experiment (19 in M1R and 14 in M2L).

### Behavioral measurements of reaction time

**Figure 3:**
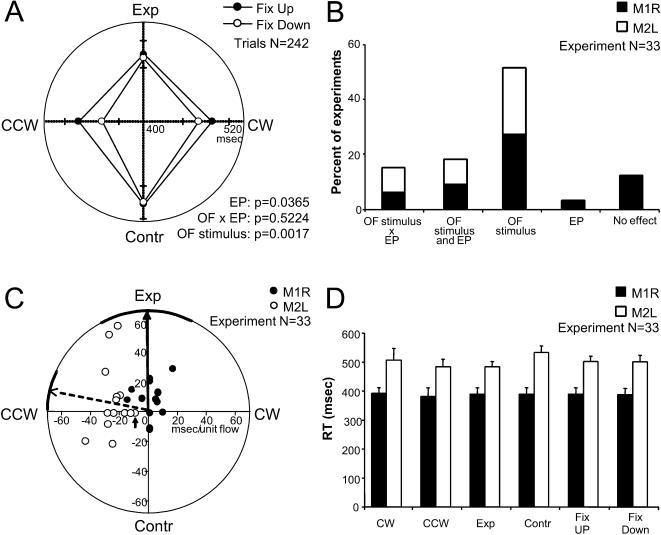
Behavioral performance in the optic flow task. A. Behavioral results from a single experiment of M2L. The reaction time is plotted as a function of the type of optic flow. Certain flows yielded shorter reaction times than others. Data are shown as upper fixation +SE, lower fixation −SE. Data set: 11-18-2002. B. Results for the ANOVA in all experiments of M1R and M2L. OF: optic flow; Exp: expansion; Contr: contraction; CW: clockwise; CCW: counter-clockwise. EP: eye position. C. Regression coefficients for both monkeys. The small arrow indicates the data from panel A. SE for each animal is indicated by the thicker line along the circumference. The mean direction for each animal is indicated by the vector arrows. The length of each arrow has no meaning. D. Average reaction times (RT)+SD for all experiments of both monkeys. FIX: fixation.

The reaction times (RT) for the different optic flow stimuli and eye positions were analyzed using a two way ANOVA (see **Methods**). Figure 3A shows RTs for a single experiment of M2L, which are significantly increased for upper fixations and radial optic flow stimuli (ANOVA, *p<0.05*). In most cases the RTs had a significant dependence on a specific optic flow stimulus as assessed by the ANOVA (Fig. 3B). Furthermore, RT were significantly different across experiments in the two monkeys (T-test, *p*<0.001).

To further quantify the dependence of the RT upon the flow and eye components, the stepwise regression (*eq.* 1) was used. The stepwise regression results in coefficients for the rotation and radial components of the RT. Figure 3C shows the circular regression coefficients for the all experiments. Each pair of coefficients defines a planar surface with the angle of the maximal slope given by Θ*_RT_* = tan^−1^(*α_rot_/α_rad_*); this angle is also the spiral space tuning angle [25]. The small arrow indicates the data represented in figure 3A. The set of resultant angles for the maximal reaction time for the two animals could be compared with a circular F-square test. The two animals have significantly different reaction times dependence upon optic flow (*p* = 0.02). Using these same angles Θ*_RT_*, a circular mean and circular standard error (CSE) were computed for each animal [24]. Typically in psychophysical studies, the shortest reaction time is used as a measure of the preferred stimulus condition. This can be computed from the Θ*_RT_* by adding 180°, as the angle is derived from a planar surface. The mean angle in M1R was 271°±27° CSE; the mean angle in M2L was 348°±9° CSE.

The regression equation also yielded coefficients for the RT dependence on eye position “*α_eye_*”. In 7 experiments the regression coefficients had negative values, meaning that downward fixations gave the longest RT; 23 experiments had values of 0 and only 3 experiments had positive values. There was no obvious breakdown between monkeys.

As for the intercept of the circular regression “β”, a clear difference was found between the two monkeys: the average RT was 317 msec±22 SD in M1R; while the average was 499 msec±16 SD in M2L, (T-test, *p*<0.001).

In summary, both monkeys' behavior, as assessed with reaction time, was dependent on the optic flow and the eye position. Each monkey had preferred navigational optic flows, inasmuch as the animal was quickest in indicating its change from structure to no-structure. Given the precise match in the speed of the dots composing the four optic flow stimuli, these preferences may represent a neural encoding selected by experience or development.

### Optical imaging of neural activity

#### Subtraction analysis

**Figure 4:**
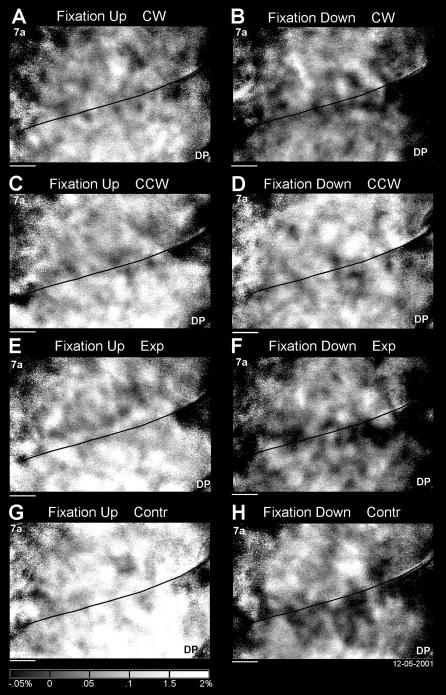
Single condition map for each optic flow stimulus at different eye positions in the right hemisphere of M1R. The baseline normalization analysis images from all correct trials in each condition were averaged together providing eight maps (4 optic flow stimuli by 2 eye positions). The average images were multiplied by “−1” to produce images whose brightness indicates expected neuronal activity. The grey scale at the bottom provides the amplitudes. The average of all maps was subtracted from each image. A. Clockwise (CW) during upward fixation. B. CW during downward fixation. C. Counter-clockwise (CCW) during upward fixation. D. CCW during downward fixation. E. Expansion (Exp) during upward fixation. F. Exp during downward fixation. G. Contraction (Contr) during upward fixation. H. Contr during downward fixation. The black lines indicate the putative border between 7a and DP. Horizontal bars: 1 mm. Data set: M1R 12-05-2001.

As in prior studies, the “difference signal” as a percent change from baseline on a trial by trial basis was computed by the baseline normalization analysis, followed by a trial rejection procedure [5], [14], [15]. Following the baseline normalization analysis, the very first step was to average all correct trials grouped by stimulus conditions providing single condition maps for a particular optic flow stimulus in a particular eye position (Fig. 4 A–H). Each optic flow stimulus has a distinctive tuning and amplitude of the reflected signal which vary with the eye position. This is evidence for a variation in the spatial distribution of activity as a function of optic flow stimulation and eye-position. This suggests that each combination of optic flow and eye position has its cortical representation. Note that the videocamera acquired images rotated with respect to the lateral view of the hemisphere (inset of Fig. 1B).

**Figure 5:**
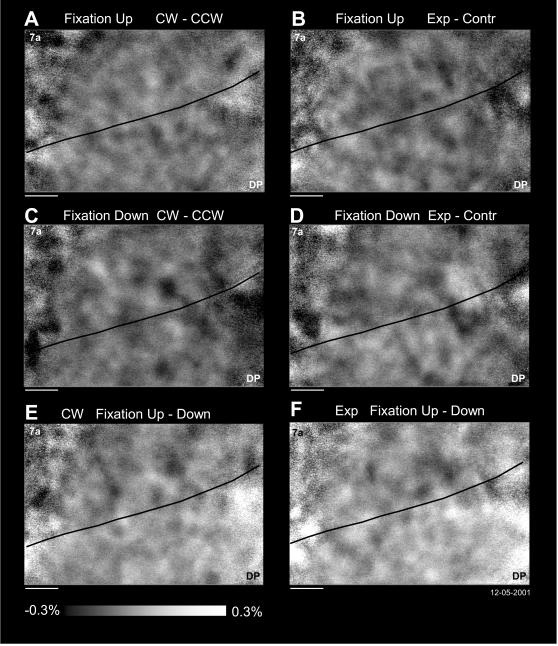
Subtraction analysis for representation of optic flow in the right hemisphere of M1R. All correct trials in each condition have been averaged together providing eight maps (4 flows by 2 eye positions). To determine the optic flow selectivity for each of the eye positions, planned comparisons were made for each opposing type of optic flow. The subtractions were performed on the images of Fig. 4. A. The average of clockwise (CW) has been subtracted from the average of counter-clockwise (CCW) for fixation in the upper part of the visual field B. Same comparison for fixation in the lower part of the visual field. C. The average of images taken during expansion (Exp) has been subtracted from the average of contraction (Contr) for fixation in the upper part of the visual field. D. Same comparison for fixation in the lower part of the visual field. E. The average of CW during upward fixations has been subtracted from the average of CW during downward fixations. F. Same comparison for Exp motion. Grey scale indicates the percentage of reflectance of the optical signal; brightness indicates increase in deoxyhemoglobin. Horizontal bar: 1 mm; data: M1R 12-05-2005.

A subtraction analysis was performed to determine the existence of a flow particular (FLO-P) tuning. This analysis provides a simple answer to the question of which stimulus pair (expansion vs. contraction or clockwise vs. counter-clockwise) evoked the maximal activation (Fig. 5). For both eye positions, the expansion map has been subtracted from the contraction map, as well as clockwise from counter-clockwise. The variation in brightness in the maps is evidence of a FLO-P topography in two eye positions (to be further quantified by the regression analyses below). The FLO-P tuning for radial and rotational stimuli during upward and downward fixations are different (Fig. 5A–D), meaning that both a certain optic flow stimulus and a particular eye position modulate the map. As confirmation of the latter, an eye position map for each stimulus has been plotted (Fig. 5E,F), where the downward fixation has been subtracted from the upward fixation in the clockwise and expansion maps. The eye position tuning with the superimposed optic flow tuning is clearly visible confirming the prior gain field results [14]. In these difference maps, area DP has more reflected light during fixation in the upper part of the visual field indicating an increased neuronal activity with upper eye positions, as expected based on prior studies [5], [14]. The darker signal in the subtraction map for area 7a indicates increased neural activity when the gaze is directed to the lower visual field. Therefore it appears from these data that optic flow and eye position signals can be simultaneously measured optically.

#### Regression analysis

**Figure 6:**
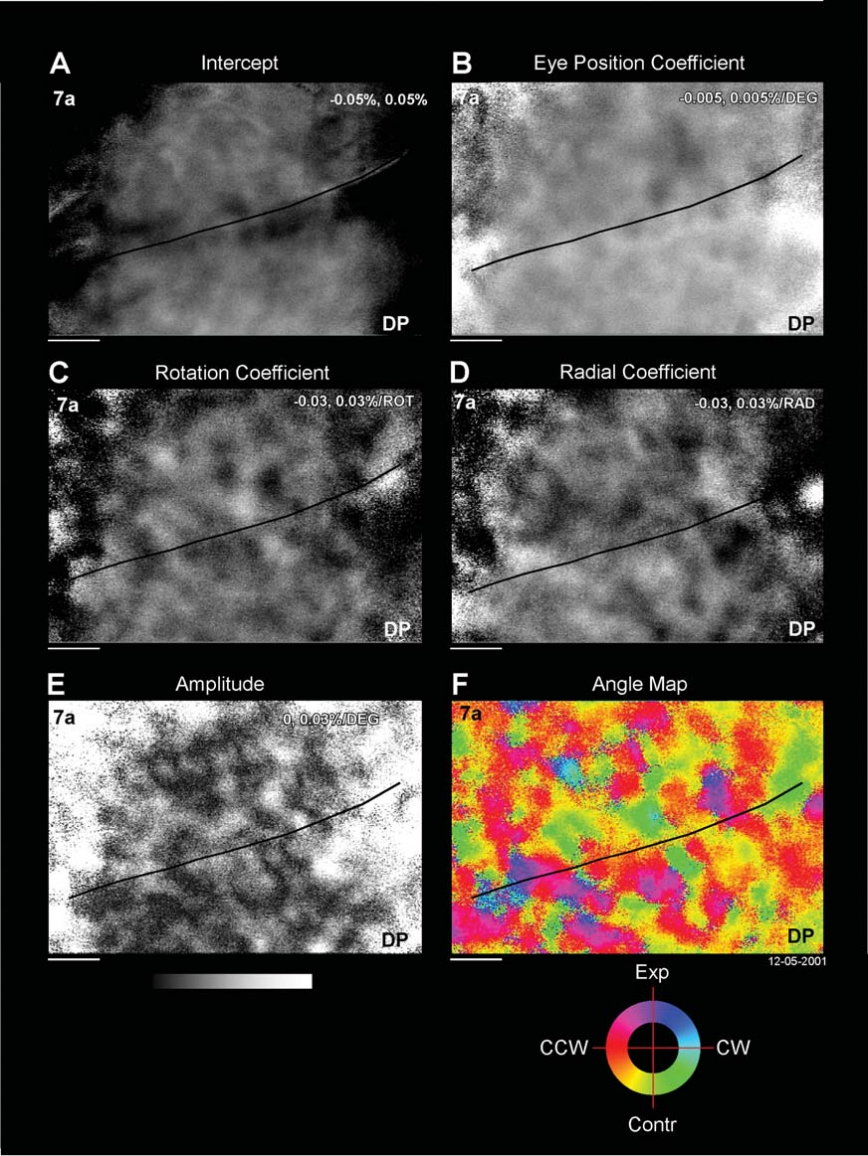
Regression analysis for representation of optic flow in M1R. Same data set as Figs. 4 and 5. Each pixel was fit as a function of each optic flow stimulus and eye position (see Methods). The resulting parameters were used to construct six parameter maps. A. Intercept shows the modeled evoked visual response for the eyes at the primary position (0, 0°) and as if there was no optic flow motion. B. Eye position map: vertical slope of the dependence of the optical signal on the eye position. C. Rotation coefficient map: dependence of the optical signal upon the radial component of the optic flow. The rotation optic flow parameter was scaled to an arbitrary value of 10 “rotation units” to represent a motion rate of 6°/sec. D. Radial coefficient map: dependence of the optical signal upon the radial component of the optic flow. The radial parameter was scaled to an arbitrary value of 10 “radial units” to represent a motion rate of 6°/sec. E. Amplitude map, root mean square of the rotational and radial coefficients. F. Angle map. The amplitude of each pixel has been converted in angular coordinates in spiral space [25]. The “spiral space” color coding represents the specific optic flow stimuli that maximally activate each pixel. The grey scale indicates the percentage of increase (bright) or decrease (dark) of the reflectance of the signal with respect to the baseline. Horizontal bar: 1 mm.

In order to quantify these effects and to separate the optic flow and the eye position components, a three-way regression analysis was used (*eq*. 2). The effects were assumed to be linearly separable. The mean optical signal was regressed with the optic flow stimulus and the eye position on a pixel by pixel basis, so to create maps of the regression parameters. The intercept map of “*β(I,J)”* (Fig. 6A) is the representation of the change in the light reflectance expected when the monkey is fixating in the primary position (0,0°) and optic flow stimulus with speed 0 is presented. The eye position map (Fig. 6B, *α_eye_*), represents the slope of the dependence of the optical signal on the eye position. Area 7a slopes have more negative values (dark area) meaning increases in the reflectance during downward fixations and DP slopes had positive values (bright area) meaning maximum reflectance for upward fixations. (Note that the sign in the model has been corrected by multiplying by −1, see **Methods**). The rotational and radial coefficient maps (Fig. 6C,D, *α_rot,_, α_rad_*) represent the dependence of the optical signal on the rotational and radial component of the optic flow. In both maps a FLO-P type tuning is evident. In the rotational coefficient map, there are certain patches tuned for clockwise rotation (bright patches) and different patches tuned for processing counter-clockwise (dark patches); the same feature is visible in the radial coefficient map, where there are patches tuned for processing expansion (bright patches) and others tuned for contraction (dark patches). The amplitude map (Fig. 6E) shows the root mean square (RMS) strength of the optic flow signals in this area of cortex.

In order to compute an angle map (Fig. 6F), optic flow selectivity was modeled using a “spiral space” approach (**Methods**, [25]). The amplitude of each pixel (I,J) has been converted in spiral space for which the color-coded angle, Θ(*I,J*), represents the type of optic flow. The eye position signal is not represented in this map.

The spiral space color coding represents the specific optic flow stimuli that maximally activate each pixel. The angle map shows many patches which covered the imaged region crossing the putative border between area 7a and DP. Typically, these patches have an approximate dimension of 1000 µm and a rounded or ellipsoidal shape and possess a clear tuning for a single optic flow stimulus or a FLO-P tuning. There is no apparent relationship to the underlying fine vasculature.

The cortical surface is flat below the blood vessel that runs between area 7a and DP in the inferior parietal lobule and there are no sulci apparent between the two areas. Interestingly, the motion patches run across this angioarchitectonic boundary, whereas the eye position signal respects the boundary (c.f. [14]). This is similar to the architecture of the attentional patches crossing the putative 7a/DP border while the eye position signals has a discontinuity there [5], [14], [15].

**Figure 7:**
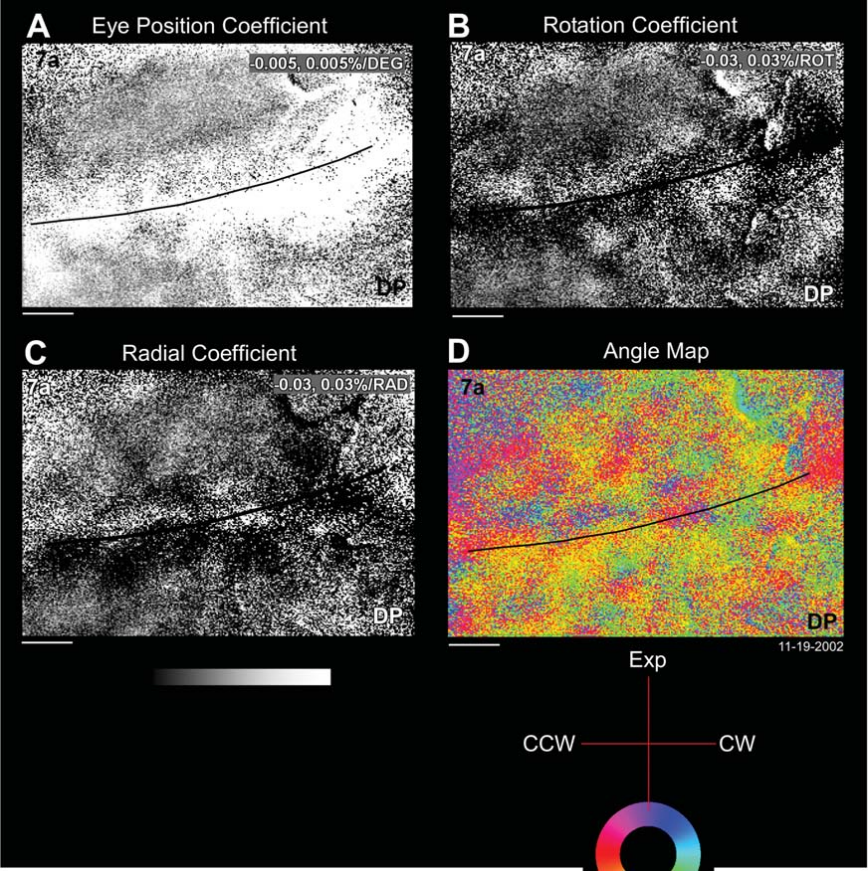
Regression analysis for representation of optic flow in the left hemisphere of M2L. A. Eye position map. B. Rotation coefficient map. C. Radial coefficient map. D. Angle map. The recording region is the same as shown in Fig. 1A. The grey scale indicates the percentage of increase (bright) or decrease (dark) of the reflectance of the signal with respect to the baseline. See legend of Fig. 6. Horizontal bar: 1 mm. Data set: M2L 11-19-2002.

These results were confirmed in the left hemisphere of the second monkey, M2L. The parameter maps (Fig. 7A–C) obtained by the regression analysis and the angle map (Fig. 7D) are illustrated. Note the recorded region is the same of that shown in figure 1. Maps recorded in M2L are comparable to those recorded in M1R. Furthermore, the optic flow representation does not seem to be ipsilateral, because all optic flows are represented across both areas.

#### Monte Carlo Analysis

The reliability of the maps (i.e. intra-day variability) was examined by a Monte Carlo analysis to determine whether stimulus independent noise was the source of the patchy architecture on a single day's data. Procedures identical to those described earlier were used [5], [14]. In short, random selections were made of half of the trials from one experiment. These trials were correctly matched with the stimulus condition (Sampled map). Alternatively the trials were randomly assigned to the stimulus conditions (Monte Carlo maps).Then the data was fit using the regression analysis of *eq*. 2. This was done 136 times resulting in 272 sets of maps.

**Figure 8:**
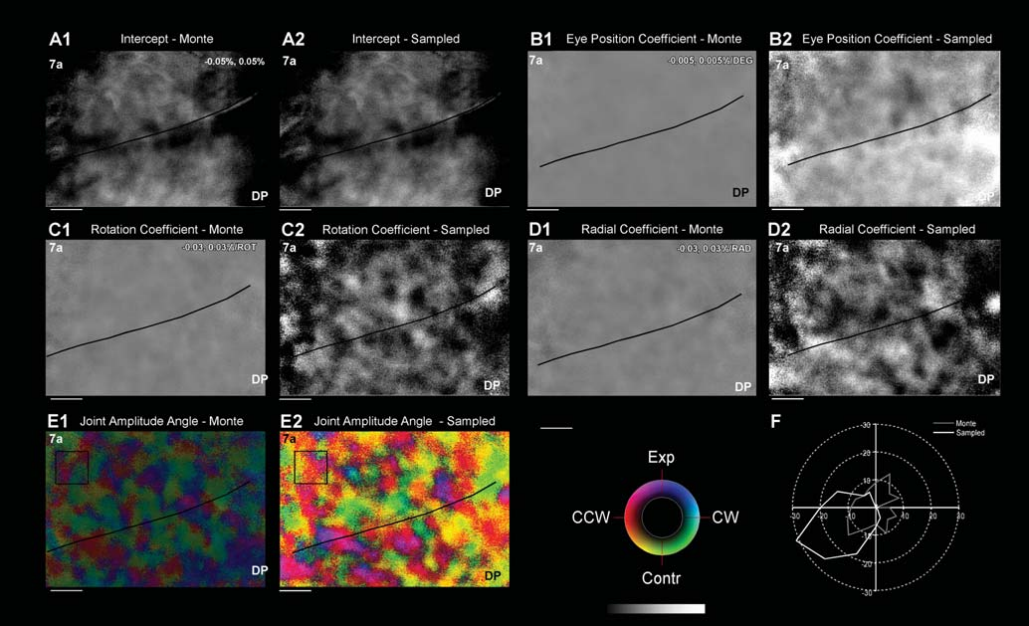
Monte Carlo and Sampled analyses. An analysis sampling the data was performed with and without shuffling the relationship between the measured optical signals and the stimulus conditions [5], [14], also see Results). Fifty percent of the trials were randomly selected and *eq. 2* was fit to the data yielding the Sampled maps. Half of the data was also reanalyzed with *eq. 2*, but the relationship between the collected data and the stimulus condition was randomized yielding the “Monte” coefficient maps. This process was repeated 136 times. Averages of the maps were computed (see Results). A1,2. Intercept parameter maps. B1,2. Eye position coefficient. C1,2. Rotation coefficient. D1,2. Radial coefficient. E1,2. Composite amplitude and angle map. The amplitude (given as a grey scale) is superimposed on the angle map. The Monte Carlo map magnitude is very small. F. Polar plot of the circular distributions angles taken from the two regions of interest (ROI) of Panel E. The radial amplitude of the distributions is the number of samples out of 136iterations. Conventions for grey scale as in Fig. 6. Horizontal bars: 1 mm. Data set M1R 12-05-2001.

The Monte parameters maps for eye, rotation and radial coefficients were averaged. No clear patch architecture was seen for any of these maps (Fig. 8B1, 8C1 and 8D1 respectively). The amplitude and the angle maps for the spiral space representation of optic flow were computed for each pixel from the length of the vector formed by the rotation and radial coefficients; these two maps were superimposed (Fig. 8E1 and E2). Whereas the Sampled map shows a strong amplitude patchy structure, there are very low amplitude patches in Monte Carlo map.

To examine the distribution of the spiral space vectors a region of interest (ROI) of 50×50 pixels was chosen (box in Fig. 8F1). For each iteration of the Monte Carlo analysis, the average radial and rotation component for the ROI was computed; from these the spiral space angle for the ROI was derived. (Note that it is necessary to compute the average angle using the component coefficients as these are angular data, see Batschelet, 1981). The distribution of these angles for the selected ROI is presented as the grey line of figure 8F. The mean vector and circular standard error of the Monte analysis data was 184.9°±69.7°; the Rayleigh test of uniformity indicate that there was not a significant deviation from a circular uniform distribution (*p* = 0.71). Thus it can be concluded that there was a uniform distribution of spiral space angles found with the Monte Carlo analysis suggesting that there observed linkage between the optic flow stimulus conditions and the patchy maps did not arise from random fluctuations.

The Sampled analysis further buttresses this argument. Following the same sampling procedure as with the Monte Carlo analysis, yet respecting the relationship between the optical data resulted in maps almost identical (Fig. 8) to those found using the entire dataset (Fig. 6). The distribution of the spiral angles in the ROI (white line of Fig. 8F) was non-uniform. The mean vector and circular standard error of the sampled data was 194.4°±3.8° (Rayleigh test for uniformity, *p*<0.01). The ROI mean angle is a good match for the colors of the angle map within the ROI.

The intercept maps for both Monte and Sampled data (Fig. 8A1, A2) are extremely similar as expected as the intercept is independent (by definition) of the eye position, rotation and radial conditions. A direct comparison is possible between the two distributions of spiral space angles for the ROI. A χ^2^ test confirmed that the two distributions are different (*p*<0.01).

Hence, it was concluded that the patches arose from a dependence of the reflected optical signal on the optic flow and the eye position and did not arise from the random fluctuations in the data. Similar results were obtained for five other maps subject to the same analysis.

#### Time course of the signal

**Figure 9:**
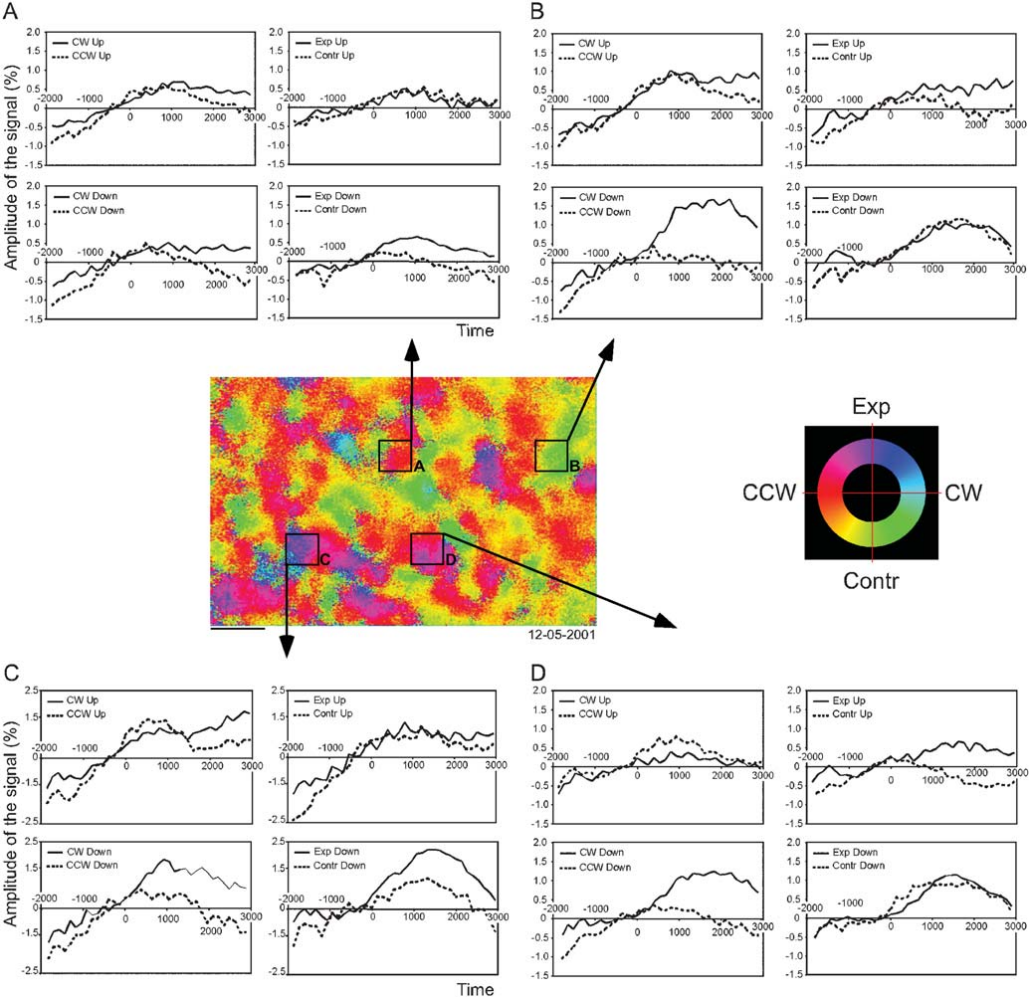
Time course of the optical signal. The time courses were averaged in four regions of interest (ROIs), two located in area 7a and two in area DP, of about 0.7×0.7 mm. The time courses were plotted for upward and downward fixations and for each type of optic flow (i.e. radial or rotational). Each ROI has been selected within a patch to show the time course of the signal relatively to a stimulus or a combination of two stimuli. Note that the vertical scale in panel C is different than panels A,B,D. −2000: onset of the fixation point; −1000 to 0: baseline window; 0: onset of the optic flow stimulus; 2000 to 3000: stimulus window. Exp: expansion; Contr: contraction; CW: clockwise; CCW: counter-clockwise. Horizontal bar: 1 mm. Data set: M1R 12-05-2001.

In order to examine the time courses across the exposed cortex, regions of interest (ROIs) of about 35×35 pixels (corresponding to 0.7×0.7 mm) were selected in both area 7a and DP (Fig. 9). The actual reflectance values are used so that positive values indicate more reflected light at 605 nm. The time courses for rotational and radial optic flow has been plotted for upward and downward fixations in the four ROIs. Typically, there was an initial negative dip in the interval −2000 to −500 msec prior to stimulus onset in these time courses. It was followed by an increase in the strength of the signal then a positive activation with a peak at about 1000 to 2000 msec. The initial dip is likely due to the first events in the task (e.g. saccade, fixation, hand movement). Variations of the percentage of the signal amplitude increment over time depending on eye position and optic flow have been seen in both animals.

#### Repeated measurements of the maps

The consistency of optic flow maps was assessed by a direct comparison of the parameter maps from the regression analysis. The maps were compared in two ways.

##### Spatial distribution of pixels

**Figure 10:**
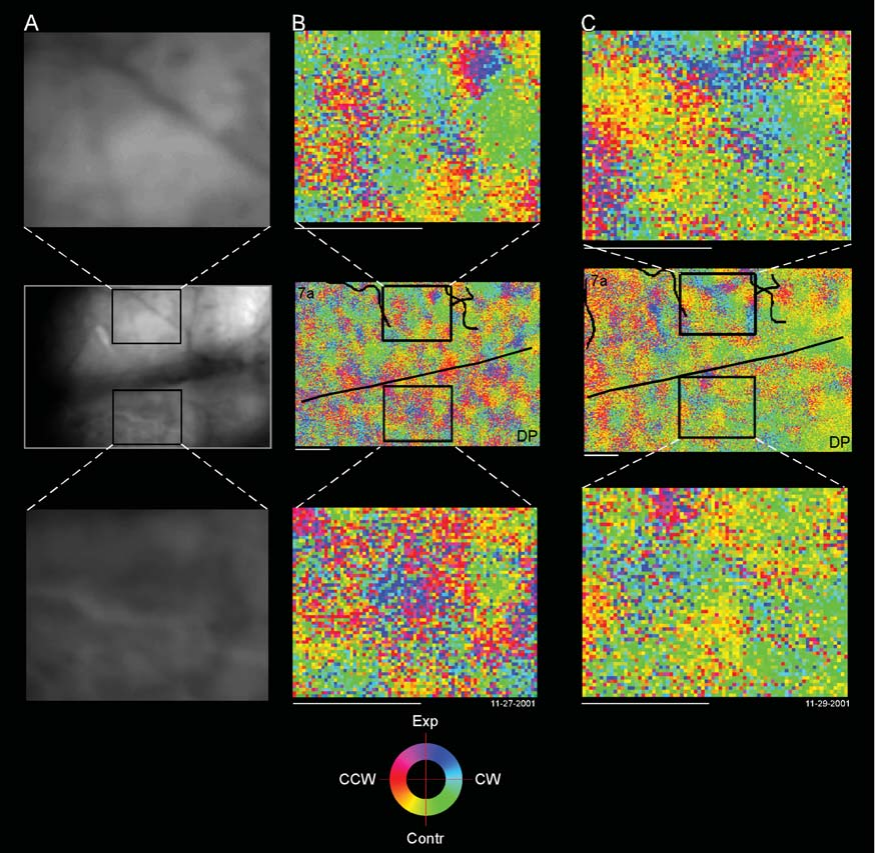
Reliability of the maps. Two different data sets are shown. A. Angioarchitectonic of area 7a and DP. The images show that the topography of optic flow is not related to the vasculature. B,C. Optical maps recorded two days apart from each other. Central portion: for each experiment a careful alignment between the map and the cortical angioarchitectonic has been made. Black lines on the optical maps indicate the drawing of superimposed blood vessels that is used as a landmark for the region of interest (ROI) selection. The squares indicate the ROIs selected in areas 7a (upper portion) and DP (lower portion). The ROIs are shown enlarged to illustrate the patchy architecture. Panel B is shown reduced so to match the resolution of panel C. Horizontal bars: 1 mm. Data set: M1R 11-27-2001 and 11-29-2001.

Patchy optic flow maps were found for every experiment in both monkeys. In order to make a comparison between experiments, optic flow angle maps were superimposed to the angioarchitectonic image always collected prior the experimental session, using the blood vessels as landmarks for the reconstruction. The same ROI was selected in both 7a and DP and then compared (Fig. 10). Each ROI is 1.8×1.4 mm. The independence of the patch structure to the underlying vasculature is supported by a comparison of figure 10A with figure 10B and C.

There was a poor match between these two maps collected three days apart (c.f. Fig. 10B and 10C). While the two have some overlap, the two maps are not tight replicas of each that might be expected if the tuning was consistent over time as demonstrated for ocular dominance [17] and retinotopy [15] in striate cortex.

##### Distribution of optic flow tuning

**Figure 11:**
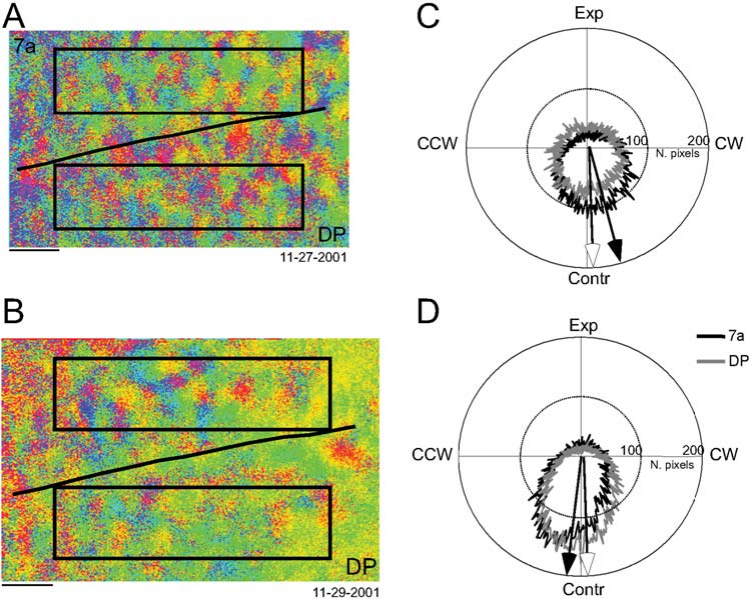
Optic flow distributions for the same data set shown in Fig. 10. A–B. Optical maps. Black lines indicate the putative border between area 7a and DP. Big squares indicate the regions of interest (ROIs) selected in areas 7a and DP for the distribution analysis. C–D. Polar plots showing the optic flow distribution of each ROI. Arrows represent the mean angle: black arrow indicates 7a, white arrow indicates DP. Panel A is shown reduced so to match the resolution of panel B. Horizontal bars: 1 mm.

For further comparison, distribution of the tuning across pixels was analyzed. The distribution of directions ψ*_7a_*(*i*),ψ*_DP_*(*i*) for the *i^th^* experiment was computed from the Θ(*I,J*) in areas 7a and DP; ROIs were 5.5×1.4 mm. For both of these examples in Fig. 11, the distributions for area 7a and DP were similar. To compare distributions across experiments, the circular means (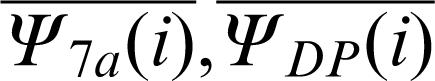) of each area for each experiment were computed (e.g. arrows in Fig. 11C,D).

**Figure 12:**
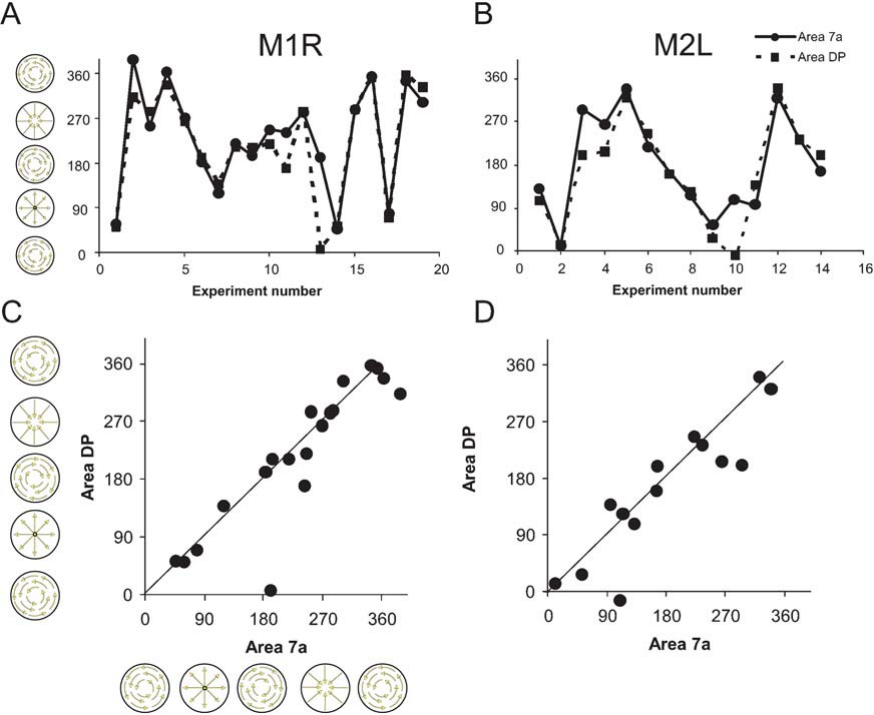
Optic flow distributions across days. A–B. Tuning for areas 7a and DP as across experiments. The angular direction indicates the mean angle taken from the distribution of optic flow tuning directions within each region of interest (ψ*_7a_*(*i*),ψ*_DP_*(*i*)). C–D. Angular correlation between the mean angular direction for areas 7a and DP. The text provides the angular correlation coefficients.

Similar measurements of the distribution 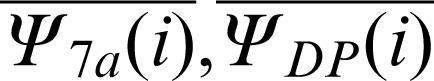 were made for all experiments using the same region of interest. In both animals, the mean tuning of each area appeared to vary across days (Fig. 12A, B). At the same time, the tuning appeared to be matched between areas 7a and DP. To determine if the tuning of each area remained constant in time across experiments, the Rayleigh test for uniformity was applied to the set of circular means for each area (e.g. 
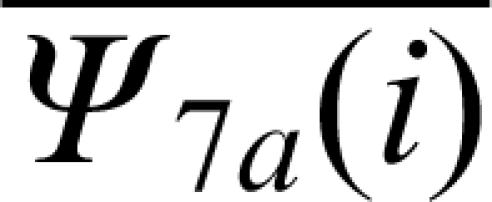
, *i = 1,n*). The distribution was always found to be uniform implying that the tuning varied across experiments. This result was found regardless of whether or not data was combined across animals. For example, across all 33 experiments, 
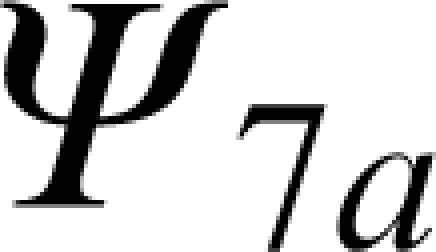
 was uniformly distributed with 281±49° CSE; the Rayleigh test for uniformity indicated a uniform distribution (*p* = 0.52). For DP, the respective measures were 262±111°, the Raleigh test was *p* = 0.88.

The apparently linear relationships between the circular means in area 7a and DP across the 33 experiments (Fig. 12C,D) were compared using the circular correlation coefficient ([24]; chapter 9). Across the 33 experiments (both animals), the 7a and DP circular means 
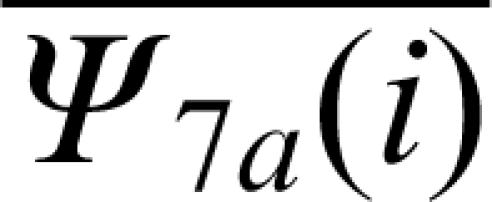
 and 
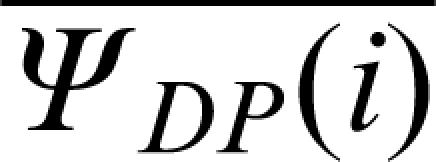
 were highly correlated (*p*<0.001), with the mean difference in angles of -11°±11° (CSE). Thus any shifts in the tuning of area 7a were accompanied by shifts in DP.

From these data, it can be concluded that the distribution of optic flow tuning changes across time, but that area 7a and DP remain correlated. Consistent properties: while the optic flow tuning was different, the eye position maps are constant and reliable across days. In every experiment, 7a was tuned for lower and DP for upper eye positions. Area 7a always shows activation during downward fixation, while area DP always shows activation during upward fixation.

### Relationship between behavior and optical flow distribution

One possibility for the variation in the distribution of circular means 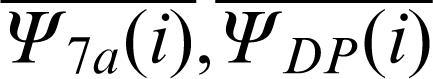 across time was that there was a consistent relationship between the behavioral preference of the monkey and the tuning of the maps (Fig. 12). The behavioral best direction computed from the reaction times (Fig. 3) was then compared to those of the optical signal's regression. There was not a significant correlation between the two using a circular linear regression for either 7a or DP (Rayleigh test, *p* = 0.43; *p* = 0.13). This result was the same whether or not the data was combined across animals. A trial-by-trial analysis for the relationship between optical data and reaction has yet to be attempted; independent component analysis may prove useful in this regard [29], [30].

#### Spatial fast Fourier transformation analysis

**Figure 13:**
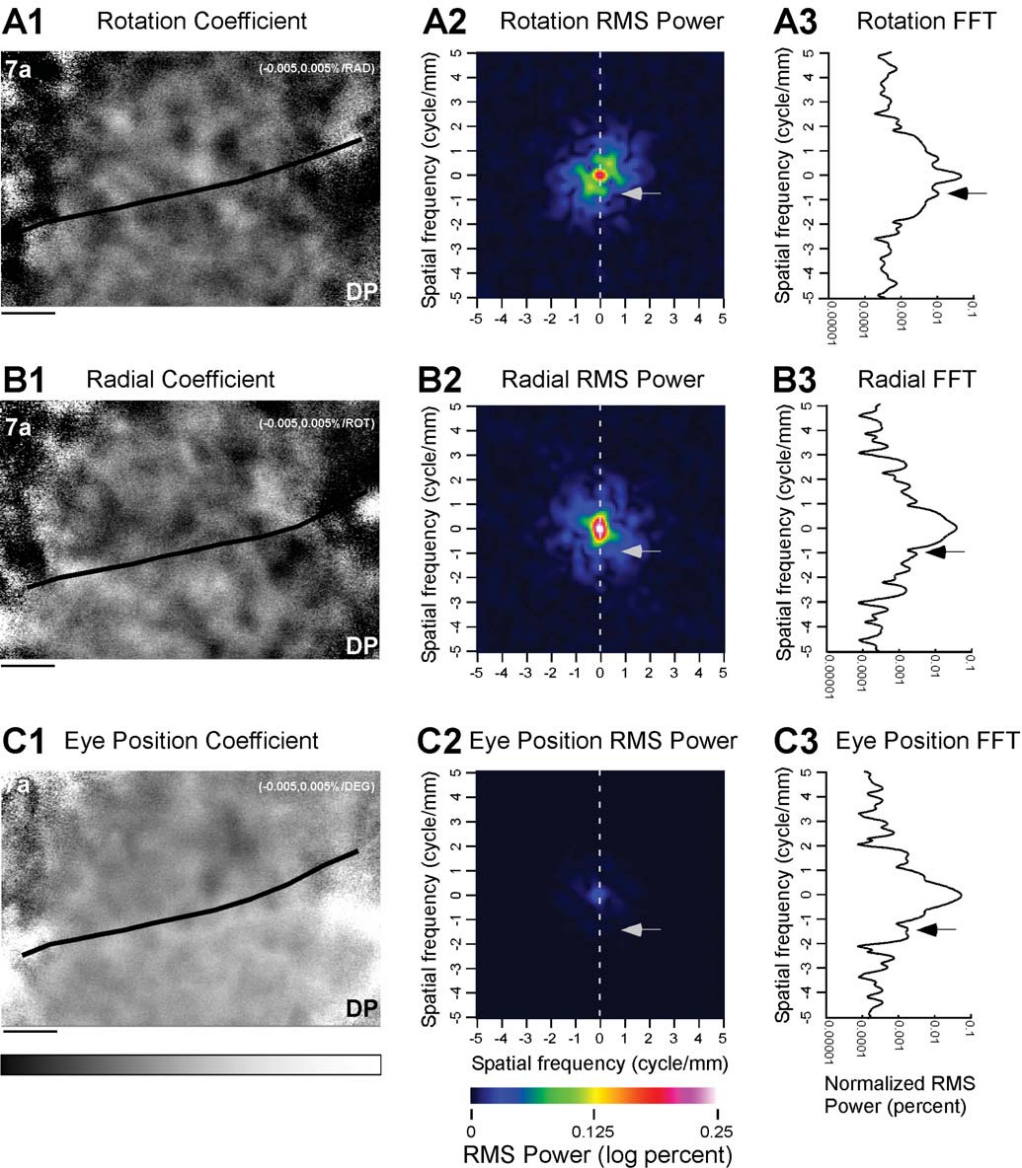
Spatial frequency analysis of parameter maps. A1. Parameter map for rotation coefficient (note that all parameter maps are the same maps shown in Fig. 6). A2. The FFT is computed (Methods) and the logarithm of the power as a function of horizontal and vertical spatial frequency is presented using a color map. The peak at the center is the DC component; there is a rapid fall-off in power as the spatial frequency increases. A3. Normalized spatial frequency dependence of the power for the rotation map. Eighteen cuts were taken through the FFT of A2. The cut taken at 90° is illustrated. For this graph, the data is normalized by the integral of the RMS power under curve. The arrow indicates the peak 0.7 cyc/mm corresponding to a spatial wavelength of 1.42 mm. B1. Parameter map for radial coefficient. B2. FFT of the radial parameter map. B3. Normalized spatial frequency dependence of the power for the radial map. The arrow indicates the peak 1.07 cyc/mm corresponding to a spatial wavelength of 930 µm. C1. Parameter map for eye position coefficient. C2. FFT of the eye position coefficient. C3. Normalized spatial frequency dependence of the power for the eye position map. The arrow indicates the peak 1.31 cyc/mm corresponding to a spatial wavelength of 760 µm. The dotted line in A–C2 represents the section shown in A–C3. Conventions for grey scale as in Fig. 6. RMS: root mean square.

While the distribution of the optic flow tuning, 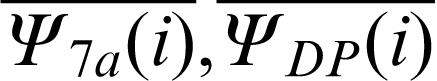, appeared to change across time, the spatial periodicity of the spatial structure appeared similar. The spatial structure of the parameter maps was quantified with spatial fast Fourier transforms for each of the three maps (rotation, radial and eye position). This was performed for all 99 maps in 33 experiments. One such example is the rotation parameter map (Fig. 13A1). The root mean square power of the spatial FFT (Fig. 13A2) shows a set of peaks around the origin ranging from about 0.8 to 1 cyc/mm. In order to select a peak value for each experiment, multiple slices were taken (see **Methods**) at 10° intervals and all were examined for the spatial frequency of the first peak. It was necessary to examine all slices since some experiments could have spurious first peaks due to noise. Figure 13A3 shows the 90° section for the rotation map of figure 13A2. A peak is seen at 0.7 cyc/mm.

**Figure 14:**
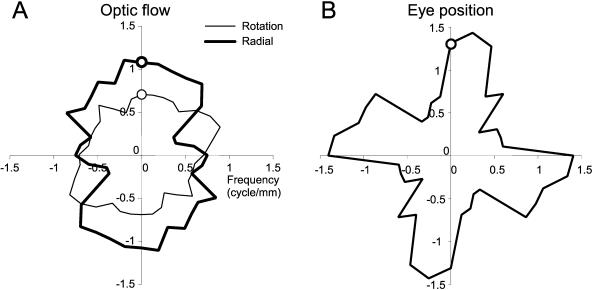
Polar plot of the spatial frequency of the peak nearest the DC peak for each angle. All 18 sections were examined in order to obtain the set of peaks consistently close to the central DC peak. A. Rotation and radial component B. Eye position component. In each plot the circle represents the data indicated by the arrows in Fig. 13.

The spatial frequency of the peak for each angle was computed (Fig. 14A, thin line). There is variation from circularity. The degree of this asymmetry varied slightly across experiments, with a mean and standard deviation of 0.69±0.1 cyc/deg. This value was close to that of the 90° measure of 0.70 cyc/deg. For the radial component (Fig. 13B2 and Fig. 14A, thick line) the mean was 0.85±0.22; the 90° value was 1.07 cyc/mm (Fig. 13B3). This analysis was also performed for the eye position parameter map (Fig. 13C1 and Fig. 14B). The first peak of the spatial frequency for the 90° cut through the data was 1.31 cyc/deg; the mean and standard deviation was 0.92±0.35 (Fig. 13C2,C3).

Thus the 90° value is close to the average of the multiple peaks. In order to choose a consistent slice though the spatial FFTs across experiments, the spatial frequency of the first peak was selected from the 90° slice in every case. (The orientation of the cortex relative to the camera was minimally changed across experiments.)

From these values, the average spatial frequency for the patches across experiments was computed. For M1R, the spatial frequency for the rotation component was 0.86±0.05 cyc/mm (mean and SE). A similar value was found for the radial component 0.90±0.04 cyc/mm, while the eye position showed higher value 0.99±0.04 cyc/mm. For M2L, the values were somewhat larger. The rotation component was 0.93 cyc/mm±0.09 SE, the radial component was 1.08±0.07 cyc/mm, and eye position was 1.12±0.1 cyc/mm. A two way ANOVA with the independent variables of “monkey” and “component type” only showed a significant difference by animal (*p*(monkey) = 0.03, *p*(component) = 0.07, *p*(interaction) = 0.92). Thus the differences across the three components were not significant, while the animals had a difference in patch size of 0.90±0.03 cyc/mm and 1.05±0.05 cyc/mm for M1R and M2L respectively. These values correspond to patch sizes of 1111 µm and 952 µm.

## Discussion

Optical imaging of the intrinsic signal assessed the representation of optic flow across areas 7a and DP while monkeys performed an optic flow motion detection task. The intrinsic signal emerges from interactions between neuronal activity and blood deoxygenation [31], primarily from the upper layers of cortex. In the present study, it was not possible to confirm the optical measurements with single unit or local field studies as penetration of the artificial dura with an electrode compromises the integrity and long-term stability of the dural implant as described elsewhere [5], [14], [15], [29]. However to date, the optical signal has been shown to match the underlying neuronal activity in every study where the comparison has been made. Hence it seems reasonable to assert that the variability of the optical signal across days would be found if long-term multisite electrophysiological recordings were made.

The functional architecture for optic flow revealed for areas 7a and DP could underlie heading perception [32]–[37]. Four orthogonal navigational optic flow selectivities were observed in agreement with electrical recordings in area 7a [8], [19].

Optical signals were recorded from monkeys performing tasks utilizing changes in the structure of navigational optic flow for a reward [38]. By the nature of the behavioral task, multiple sensory, cognitive and behavioral events occurred during the recordings. Here across the eight conditions, only optic flow and eye position were systematically varied, making the optical signals statistically independent of events that are attentional, intentional, etc. This design provided the statistical power necessary to extract signals from the low signal-to-noise in optical imaging.

The eight conditions sampled navigational optic flows (clockwise, counter-clockwise, expansion, contraction) under two angles of gaze. The optic flows were constructed with precisely the same velocity distributions, number of dots, diameter and point life [18]. Therefore the measured changes in the intrinsic signal were directly attributed to the type of optic flow and not to superfluous stimulus differences such as velocity distribution.

An alternative explanation for the tuning of the cortex to the optic flow stimuli is the local motion. Single unit recordings in associational cortices, such as the middle superior temporal (MST) area, the anterior superior temporal polysensory (STPa) area and area 7a [18], [20], [25], [39]–[42], suggest that the responses to global optic flow cannot be explained in terms of local motion fields. However, the optic signal reported here may reflect different physiological sources (e.g. neuropil vs. soma) and so the contribution of local motion fields cannot be completely discarded. Studies in which patches of translation motion selectivity is contrasted with optic flow selectivity can examine this question further.

The inclusion of the two eye positions in the experimental design served two purposes. First, the relationship between *both* the eye position and optic flow could be determined. Second, and importantly, the eye position dependence on the optical signal provided an internal control in each experiment [5]. Upper and lower eye positions (as opposed to horizontal positions) were selected as evoking larger optical signal changes. Thus, the experimental design obviated dependence upon electrical recordings. In our hands, repeated electrical recordings damaged the artificial dura and underlying cortex, making longer term optical measurements tenuous [5], [14], [15].

### Time course of the optic flow responses

The time courses of the intrinsic signals demonstrate the differential effects of the types of optic flows. Within patches, different amplitude signals can be observed based upon the type of optic flow presented. In general for these time course, the amplitude of a particular optic flow response dominates throughout the image presentation. This suggests that the optic flow tuning is constant throughout the recording period, matching the electrophysiological recordings of optic flow responses in single neurons [8], [20], [26]. This consistency between the two measurements is supportive that the optical responses reflect the neuronal responses.

### Functional architectures, optic flow and gain fields

Gain fields were observed optically confirming our group's earlier findings. Two architectural features in the inferior parietal lobule were novel. First, the reflectance of 605 nm light from DP cortex varied with optic flow as shown by single condition maps, subtraction maps, and regression analysis. This novel finding of optic flow selectivity in DP implies selectivity to optic flow in neurons; such single units have not been reported. Until now, in the inferior parietal lobule, optic flow responsiveness only has been described in 7a neurons [8], [19], [26], while, to our knowledge, DP neurons have been tested only with classical visual stimuli and eye-movement tasks [10], [43]–[45].

The second novel finding is the patchy architecture for optic flow embedded within the gain fields. The patchy architecture is for FLO-P tuning, i.e. particular optic flow stimuli maximally activate a cortical region. The patchy structure of optical response to navigational flow is not surprising. Lateral intraparietal area (LIP) , STPa and MST have been reported to carry such signals [1] and have patchy anatomical projections to inferior parietal lobule [43], [46]. Other evidence for columnar representation of optic flow is for area MST from double-label 2-deoxyglucose or reconstructed electrophysiological methods [47]–[49]. The current work extends these critical studies into a full two-dimensional map of optic flow across the inferior parietal lobule. The periodicity of the optic flow patches was 950 to 1100 µm in the functional maps reported here for DP and 7a. This differs from the MST findings which demonstrated clusters of ∼500 µm. The difference in the “cluster” and “patch” sizes may arise from technical differences between electrical and optical recordings or may represent true areal differences.

### Characteristics of the inferior parietal lobule functional architectures

There were three main features of the functional architecture for optic flow and eye position under these behavioral conditions.

#### Gain field border

First there was a border between upper and lower gain fields that remained at the same location across all experiments described here, and in the gain field [14] and attention studies [5], [14], [15]. This border consistently was found at the blood vessel running on the dorsal surface. The consistent border has been invoked to indicate a dominance of anatomical connectivity in the establishment of the cortical architecture [5], [14], [15].

#### Patchy structure

The second feature for the functional architecture under these behavioral conditions is a patchy structure for flow and gain fields. The spatial frequency of the patches was consistent across the thirty-three experiments in two monkeys with a spatial periodicity of 950–1100 µm. The flow patch size was somewhat larger those of the attentional study (∼860 µm; [5]). Both flow and attentional patches crossed the putative border between area 7a and DP. A better understanding of the relationships between the circuits of the two dynamic representations requires experiments jointly manipulating attention and flow.

For area 7a and DP, the range of optic flow tuning in the patches was evaluated for ∼8 mm^2^ regions of interest. Within each experiment, the average direction was correlated between areas 7a and DP, with very small differences found between the two areas. This suggests that the tuning of flows between the two areas was linked whereas eye position signals were different.

#### Inter-day variability

The third feature of the functional architecture is variability in the location of patches for optic flow tuning that were embedded in the gain field map; the patches were not stationary. Across the thirty-three experiments, the patches of the optic flow functional architectures varied in location. This supports the hypothesis put forward by Raffi and Siegel [5] that the dynamic patchy functional architecture reflect ongoing activity.

The maps were not good replicas across days. It is highly unlikely that the variability derived from experimental issues. All experiments were performed under the same conditions (e.g. intensity of cortical illumination, stimulus parameter, and cortical depth of imaging).

The Monte Carlo analysis demonstrated that the presence of the patches did not arise from random fluctuations in the experimental setup or the data (c.f. [5] and [14]). The patches were correlated with the stimulus conditions. In an earlier study [14] that examined gain fields using a single expansion flow as a test stimulus, patches were not found. This suggests that the patches arise from the multiple flow fields being tested. Thus there was good consistency within a day. The maps changed after replacing the animal into his housing for one or more days.

Further quantitive analysis of the variability examined the distribution of optic flow tuning across time. The statistics of the distributions 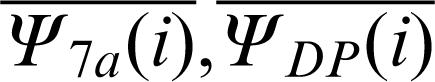, indicate that there is variability across days within area 7a and within DP.

One possible explanation for this variability was the animal's behavioral performance. It was possible for each animal to determine the type of optic flow for which changes in structured motion were easiest to detect on each experimental day. Overall across days one animal preferred expansion optic flow while the other preferred counterclockwise flow. The day-by-day behavioral and optic measurements were tested for angular correlation. This analysis failed to show a significant correlation indicating that at least the monkey's behavioral performance on each day was not a good predictor of the optical maps.

Other possibilities for the source of the variation in time may be experiential events, internal state, as well as task demands (e.g. difficulty). While the source of the variability eludes us, the existence of the variability under these conditions in the distribution of tuning in area 7a and DP further supports the hypothesis that ongoing activity can alter the functional architecture, as proposed for the retinotopy and attentional patches [5], [15].

The variation in the tuning of areas 7a and DP over days is in contrast to the close match of tuning between area 7a and within each day (∼11°). The lockstep in tuning between area 7a and DP is consistent with a global mechanism providing an organizational signal across the ∼30 mm^2^ area imaged here. Candidates are long range horizontal connected circuits, spatially divergent feedback from layer II of frontal areas, direct interactions between area 7a and DP, and sub-cortical projection from locus coeruleus basal forebrain and basal ganglia [43], [50]–[54].

### Possible mechanisms for the variation of topographical representations

Topographical representations in parietal cortex [5], [14], [15] and a number of other cortical areas [55]–[59] are plastic. While these studies encompass a range of cortical functions, common mechanisms have been proposed for stationarity and variability of cortical architectures [60], [61]. It appears that neurons are constantly modulated by feedback and lateral interactions to provide a temporally short-lived but useful representation of the sensory input, behavioral state, and motor plans. Sub-cortical projections from elements such as the cholinergic basal forebrain or the locus coeruleus noradrenergic systems may permit dynamic modulation by altering pre-synaptic and post-synaptic plasticity at the level of receptors which translate into self-organizing global population behaviors [58] via integration in dendrites. We propose these global behaviors are observed as patches in the inferior parietal lobule.

Recent studies on human subjects suggest that the functional organization of the sensory cortex can dynamically change according to the task [62], [63] Furthermore, several experiments demonstrated that long-term perceptual training or attentional shifts reorganize human somatosensory cortex [64]–[68]. Results of the present study are in agreement with these cited papers and could clarify some neuronal mechanisms that could underlie plasticity by providing observations at a scale much finer than fMRI.

### On the functional definition of cortical areas

Multiple criteria define a cortical area [69]; one is physiological responsiveness. A hypothesis of four cortical networks across areas 7a and DP can be posited. Two of these architectures (gain, retinotopy) vary gradually with cortical location albeit with dramatically distinct representations between 7a and DP. Two dynamic architectures (attention, optic flow) involve embedded patchy representations extending across the putative border between 7a and DP. Traditional static cytoarchitecture, myeloarchitecture, anatomical connectivity, and immunohistochemistry of parietal correlate, *in part*, with our putative segmentation of DP and 7a [43], [46], [70]–[73].

If association cortex is as malleable as our studies indicate, rapid structural and biochemical and genetic changes should occur in parallel. Hence, affirmation of the concept of dynamic functional architectures underwritten by ongoing activity may require an extension of Van Essen's criteria. Perhaps with the assistance of novel genetic approaches [74], [75], detailed *in vivo* exploration can permit the incorporation of mechanisms of both static and dynamic functional architectures that define association cortical areas and ultimately support complex cognitive function.
